# Twitter Sentiment About the US Federal Tobacco 21 Law: Mixed Methods Analysis

**DOI:** 10.2196/50346

**Published:** 2023-08-31

**Authors:** Page D Dobbs, Allison Ames Boykin, Nnamdi Ezike, Aaron J Myers, Jason B Colditz, Brian A Primack

**Affiliations:** 1 Health, Human Performance and Recreation Department University of Arkansas Fayetteville, AR United States; 2 Education Statistics and Research Methods University of Arkansas Fayetteville, AR United States; 3 Division of General Internal Medicine University of Pittsburgh School of Medicine Pittsburgh, PA United States; 4 College of Public Health and Human Sciences Oregon State University Corvallis, OR United States

**Keywords:** social media, Twitter, Tobacco 21, mixed methods, tobacco policy, sentiment, tweet, tweets, tobacco, smoke, smoking, smoker, policy, policies, law, regulation, regulations, laws, attitude, attitudes, opinion, opinions

## Abstract

**Background:**

On December 20, 2019, the US “Tobacco 21” law raised the minimum legal sales age of tobacco products to 21 years. Initial research suggests that misinformation about Tobacco 21 circulated via news sources on Twitter and that sentiment about the law was associated with particular types of tobacco products and included discussions about other age-related behaviors. However, underlying themes about this sentiment as well as temporal trends leading up to enactment of the law have not been explored.

**Objective:**

This study sought to examine (1) sentiment (pro-, anti-, and neutral policy) about Tobacco 21 on Twitter and (2) volume patterns (number of tweets) of Twitter discussions leading up to the enactment of the federal law.

**Methods:**

We collected tweets related to Tobacco 21 posted between September 4, 2019, and December 31, 2019. A 2% subsample of tweets (4628/231,447) was annotated by 2 experienced, trained coders for policy-related information and sentiment. To do this, a codebook was developed using an inductive procedure that outlined the operational definitions and examples for the human coders to annotate sentiment (pro-, anti-, and neutral policy). Following the annotation of the data, the researchers used a thematic analysis to determine emergent themes per sentiment category. The data were then annotated again to capture frequencies of emergent themes. Concurrently, we examined trends in the volume of Tobacco 21–related tweets (weekly rhythms and total number of tweets over the time data were collected) and analyzed the qualitative discussions occurring at those peak times.

**Results:**

The most prevalent category of tweets related to Tobacco 21 was neutral policy (514/1113, 46.2%), followed by antipolicy (432/1113, 38.8%); 167 of 1113 (15%) were propolicy or supportive of the law. Key themes identified among neutral tweets were news reports and discussion of political figures, parties, or government involvement in general. Most discussions were generated from news sources and surfaced in the final days before enactment. Tweets opposing Tobacco 21 mentioned that the law was unfair to young audiences who were addicted to nicotine and were skeptical of the law’s efficacy and importance. Methods used to evade the law were found to be represented in both neutral and antipolicy tweets. Propolicy tweets focused on the protection of youth and described the law as a sensible regulatory approach rather than a complete ban of all products or flavored products. Four spikes in daily volume were noted, 2 of which corresponded with political speeches and 2 with the preparation and passage of the legislation.

**Conclusions:**

Understanding themes of public sentiment—as well as when Twitter activity is most active—will help public health professionals to optimize health promotion activities to increase community readiness and respond to enforcement needs including education for retailers and the general public.

## Introduction

Tobacco 21 is a tobacco control law that raises the minimum legal sales age (MLSA) of tobacco products to 21 years. Since the enactment of a local Tobacco 21 policy in Needham, Massachusetts in 2005 [1], more than 570 municipalities and 42 states in the United States have passed Tobacco 21 policies [2], and more than 200 of these policies were passed between 2018 and 2019 [3]. Such widely accepted policy diffusion met what may be considered a tipping point on December 20, 2019, when the US congress passed a federal Tobacco 21 bill into law that went into effect immediately [4]. Benefits of Tobacco 21 laws include reductions in current cigarette use among youth [1] and those aged 18-20 years [5] who report previous cigarette use [6]. Additionally, the Institute of Medicine suggests the federal law could save nearly a quarter of a million lives by preventing premature death associated with tobacco use [7].

Public support may have contributed to the rapid enactment of local and state Tobacco 21 policies. Studies conducted between 2013 and 2017 via phone-based questionnaires and web-based surveys found adults and adolescents broadly supported these policies, believing them to be effective methods of reducing youth access to tobacco products [8-11]. However, polling studies, such as these, rely on closed-ended measures that may not capture all beliefs about and feelings toward these policy initiatives. Also, the cross-sectional nature of prior studies limits the ability to determine changing views toward local or state policy leading up to enactment and implementation. Examination of time-stamped data, such as social media–based data, may allow researchers to explore public opinion and sentiment toward tobacco control policies (eg, Tobacco 21) in a way that complements traditional data collection methods. Thus, data leading up to the enactment of this law may help explain temporal trends regarding public sentiment toward tobacco control policies that may influence community readiness for policies and public acceptance of adoption of laws once enacted. Although local community readiness may not directly influence policy change at higher levels of the government (eg, federal government) or create top-down change, it can influence change at lower levels of the government, creating a grassroots effort that can diffuse to parallel or higher levels of the government [12]. Thus, understanding trends about policy discussions may help forecast adoption of a law or potential pitfalls after adoption, based on the sentiment of those affected by the policy change.

Social media data have illuminated perceptions and sentiment about various nicotine and tobacco related topics, including e-cigarettes [13-16], hookah tobacco smoking [17,18], youth and young adult tobacco use [19,20], and tobacco product branding [21]. However, relatively few studies have explored sentiment toward tobacco control policies [22-28]. Because Twitter has been recognized as the main social media platform for policy-related discussions regarding tobacco products [13], it is not surprising that these studies have advanced understanding of the current use of tobacco products and have successfully curtailed tobacco and nicotine use. For example, acknowledging Twitter sentiment about local regulation of e-cigarettes in Chicago, Illinois one week before the city council voted on this addendum allowed public health professionals to respond to antipolicy sentiment by posting a call to action that was shared widely through local networks to policymakers [22]. Further, Twitter sentiment data about a proposed tobacco control measure that failed to pass (increasing tobacco tax) in California, indicated public support on social media declined leading up to the vote [24]. Although supportive or “pro” Twitter chatter regarding this California bill outnumbered the negative tweets both early in the campaign and as the proposition approached a public vote, the pro tweets were characterized as weaker messages or failed to clearly tie the prevention message to young audiences [24]. Such information can be important after policy initiatives to provide lessons learned for others to consider.

There is a dearth of literature exploring discussions about Tobacco 21 on Twitter [25,26]. To date, research suggests misinformation about Tobacco 21 circulated via news sources on Twitter [25], and sentiment about the federal law was associated with particular types of tobacco products (eg, those about e-cigarettes were more supportive of the law while those about cigarettes were more likely to include anti or neutral sentiment) and included discussions about other age-related behaviors (ie, military enlistment, alcohol use, legal voting, and purchasing a firearm) [26]. Although sentiment on Twitter toward the law has been explored [26], underlying themes of sentiment toward the federal Tobacco 21 law has not. Although text mining approaches can provide a broad exploration of more data about a topic [23], machine learning algorithms created to identify sentiment are limited by accuracy flaws and often require smaller annotated data sets to train these deep learning classification models [29]. Thus, examining tweets annotated by humans is foundational to ensure the sentiment is relevant to the topic.

Additionally, past research about Tobacco 21 discussion on Twitter has not examined the volume patterns of Twitter chatter (number of tweets over time) before the law was enacted. Similar to prior research that explored Twitter chatter about tobacco control policies over time [15,24,27], we sought to understand how the federal Tobacco 21 law was discussed on Twitter before it successfully passed. Understanding this can help provide meaningful recommendations to those developing communication campaigns about emerging tobacco control policies. Therefore, the purpose of this study was to explore Twitter sentiment about Tobacco 21 in the final months leading up to the enactment of the US federal law by examining themes within tweets identified to be pro-, anti-, and neutral policy discussions. Further, we examined volume patterns (number of tweets) of Twitter discussions about this policy before it was passed and integrated qualitative findings to help explain peak patterns.

## Methods

### Study Design

Twitter messages (ie, tweets) consist of brief text (up to 280 characters and emojis) with media and links. Due to the brevity and richness of tweets, and the popularity of the Twitter platform in the United States [30], several studies have used Twitter data for surveillance of public perception of tobacco-related topics [16,20,23,31-34]. We used a mixed-methods study design to assess the quantitative breadth and qualitative depth of tweets related to the federal Tobacco 21 policy. We identified tweets during this time frame due to the public attention to this issue during the last months leading up to the enactment of the federal Tobacco 21 law, approved by congress and signed by the US president on December 20, 2019.

### Data Selection

Data were collected using the Real-Time Infoveillance of Twitter Health Messages (RITHM) open-source software for Python (Python Software Foundation) [35,36]. The RITHM software was developed by a team of researchers at the University of Pittsburgh beginning in 2014 [35], was released on GitHub in May 2017, and was retired in September 2022 due to changes in Twitter’s application programming interface. While RITHM was actively running on our institutional data server, we were able to collect all publicly available tweets that included specific keywords in their text. For this study, we obtained tweets matching tobacco-related keywords (ie, *tobacco*, *nicotine*, *cigarette*, *cigarettes*, *e-cigarette*, *e-cigarettes*, *vaping*, *vape*, *vapes*, *vaper*, *vapers*, *vaped*, *ecig*, *ecigs*, *e-cigs*, *e-cigs*, *cig*, and *cigs*) [35]. To narrow our temporal focus to a time frame where the Tobacco 21 legislation was garnering public attention, we initiated data collection on September 4, 2019, and continued through the end of the calendar year. Data collection was relatively stable over this time, with the exception of December 7-13, when the data server was offline for maintenance. We then filtered by keywords relevant to the Tobacco 21 policy (ie, *18*, *21*, *age*, *buy*, *mcconnell*, *mitch*, *purchase*, *t21*, and *tobacco21*). Some tweets were included due to time-sensitive topics, such as the name of Senate Majority Leader Mitch McConnell (a Republican from Kentucky) because he championed the first Tobacco 21 bill filed in 2019. The original search found 615,574 tweets matched with the search terms. After removing retweets (ie, resharing of existing tweets), this sample was reduced to 231,447 unique tweets. Prior Twitter-based studies recommend methods of reducing the sample to a feasible number for analysis while maintaining integrity of the data that include randomly selecting tweets or stratifying data from a 1%-5% sample [31,35]. For this study, a random 2% subsample (n=4628) of unique tweets, stratified by frequency of tweets per day, was selected based on a reasonable number of tweets for human annotation.

### Coding Process

Using a deductive coding procedure, 2 independent coders (initials redacted) were trained by a senior researcher (initials redacted) to categorize tweets according to variables identified in prior Twitter-related studies: relevance, news, and sentiment. For this study, relevance was operationalized as tweets related to the federal Tobacco 21 policy by removing tweets that referenced a state or local law, and news was operationalized as messages that originated from a news outlet or related to a news story. Tweets identified as discussion about a state or local Tobacco 21 policy were coded as such and labeled to not be relevant for this analysis due to varying themes that focused on policy provisions not including in the federal law (eg, military exemptions, preemption, and grandfathering). Sentiment was categorized as messages with either generally favorable (pro), unfavorable (anti), or neither (neutral) opinions of the Tobacco 21 policy; operational definitions have been cited elsewhere [37]. Sentiment context derived from both text and images (eg, emojis, pictures, memes, and videos) portrayed in the tweet; thus, coders were provided a link to the original tweet to review all visual context and links to external sources (eg, news articles) when applicable.

Coders were provided with the tweet text, a link to the web-based version of each tweet, and if the tweet was in response to another account’s tweet, this tweet was provided as well. Using this information, coders were trained to identify tweets relevant to Tobacco 21 policies using an iterative process of coding 200 tweets during week 1 and 500 additional tweets during weeks 2 through 9. Using Cohen κ as a measure of inter-rater agreement [38], which indicates the proportion of agreement after accounting for chance agreement, coders had strong (κ>0.80) for all variables after the fourth week of coding (κ values ranged from 0.855 to 0.982). Data had been used in prior research [25,26]; however, they were annotated by separate researchers using an updated codebook.

### Qualitative Analysis

As conducted in prior social media–based mixed methods research [31], the qualitative breadth of data were explored using a phenomenological process that acknowledges the research teams’ subjective and preconceived bias while expanding meaning from smaller codes to a broader understanding of the data [39]. After all data were coded, the 2 coders and senior researcher (lead author) met to compare notes about emergent themes, or similar ideas that emerged from the data [40]. For example, all pro-, anti-, and neutral policy tweets were reviewed, and using a thematic analysis, themes per sentiment category were identified. Once the research team came to consensus on themes and representative tweets, a thematic narrative was contextualized to explain the quantitative results. The narrative was also used to update the operational definitions in the codebook, and all data were recoded to total frequencies of identified themes. Coders indicated strong agreement for the emergent themes (κ values ranged from 0.861 to 0.960). Representative tweets were included in Table 1, and paraphrased tweets and aggregated context were reported to protect Twitter users’ identities, as advised by prior research [41].

**Table 1:** Operational definitions and example tweets per theme of pro-, anti-, and neutral policy toward Tobacco 21 (N=1113).

Sentiment and theme		Total count	Operational definition	Example tweet
**Propolicy (167/1113, 15%)**				
	Sensible regulation	33	Tweet indicates support for Tobacco 21 as long as the products or flavors are not banned.	“Sensible regulation! Adults want flavors. T21. Online ban. Ban juul. Ban gas station/C store sales. Make vape stores like liquor stores! Ban pod based systems. Prohibition=black market poison.”
	Protection for youth and young adults	46	Tweet states or describes how the law will protect youth, young adults, kids, high school students, or those 18-20 years old.	“We must protect our youth by lobbying laws which require a person 21 years old and over to purchase tobacco products.”
**Antipolicy (432/1113, 38.8%)**				
	Addiction among young audiences	159	Tweet mentions that the law will make it difficult for youth, young adults, kids, high school students, or those aged 18-20 years who are addicted to or dependent on nicotine or named tobacco product.	“Does it really matter if you get addicted at 21 rather than 18? Is it really going to change anything? If you are an adult at 18 and can go to war I think you are old enough to smoke if that is what you want to do.”
	Sarcasm or criticism about law	61	Tweet appears to use mock or make an ironic statement.	“Thank god they’re handling the important things...(Tweet in response to a news article about Tobacco 21).”
**Mixed sentiment—ways to evade law**				
	Antipolicy	42	Tweet describes methods for those under 21 to obtain tobacco.	“So you say making it illegal for 18-21 year old to buy legal vapor products will save lives? Now they will just go to their “dealer” since they can no longer buy in a store.”
	Neutral policy	26	Tweet describes methods for those under 21 to obtain tobacco.	“Don’t call me if you under 21 to buy tobacco cause I ain’t answering.”
**Neutral policy (514/1113, 46.2%)**				
	Political	143	Tweets that mention a political figure or party.	“Had to turn down a customer buying tobacco products today because he was 18. I was like come back in 3 years and blame the government baby boy I’m sorry I don’t make the rules. I just make the money.”
	News	179	Tweet is a news article about Tobacco 21 or provides a link to a news article about the bill or law.	“Bill to raise tobacco age has unlikely allies: Altria, Juul.”

### Quantitative Data Analysis

Once coded, data were explored via descriptive statistics using R statistical software (R Core Team) and trends were visualized using the ggplot2 package. Frequencies of tweets were first assessed per day of the week, to examine if Twitter discussions about Tobacco 21 occurred more often on certain days of the week. Next, frequencies of the tweets coded as pro-, anti-, and neutral policy were reported to determine the most common sentiment tone. Finally, the temporal trends of tweets about Tobacco 21 were examined using timestamps and tweet content to explore trends in social media communication about this policy leading up to the enactment of the federal law.

### Mixed Methods Analysis

Twitter data have been noted for their optimal ability to be used for mixed methods analyses due to the quantity of data that allow for the quantitative exploration of depth while also providing a qualitative content analysis, enhancing the breadth of understanding about a topic [31]. The distinction between mixed methods, as opposed to the use of multiple methods, is the point of convergence, or the integration of the 2 methodologies [42]. In prior research that leveraged social media to explore vaping-related topics, data were integrated throughout the analysis process, with emphasis quantifying the phenomenological context of Twitter data [31]. For this study, we used a similar convergent parallel approach due to the phenomenological approach of exploring a particular topic “sentiment toward Tobacco 21” on Twitter. Thus, the qualitative research was first contextualized into codes, and then the codes were quantified for analysis. Finally, the researchers reintegrated the findings by exploring the narrative that helped to explain the context of Tobacco 21 discussions during times where Twitter chatter peaked.

### Ethical Considerations

This was an observational study of public discussion about Tobacco 21 on the Twitter social media platform, exempt by the University of Arkansas institutional review board (protocol # 2203394304).

## Results

### Overview

**Figure 1:**
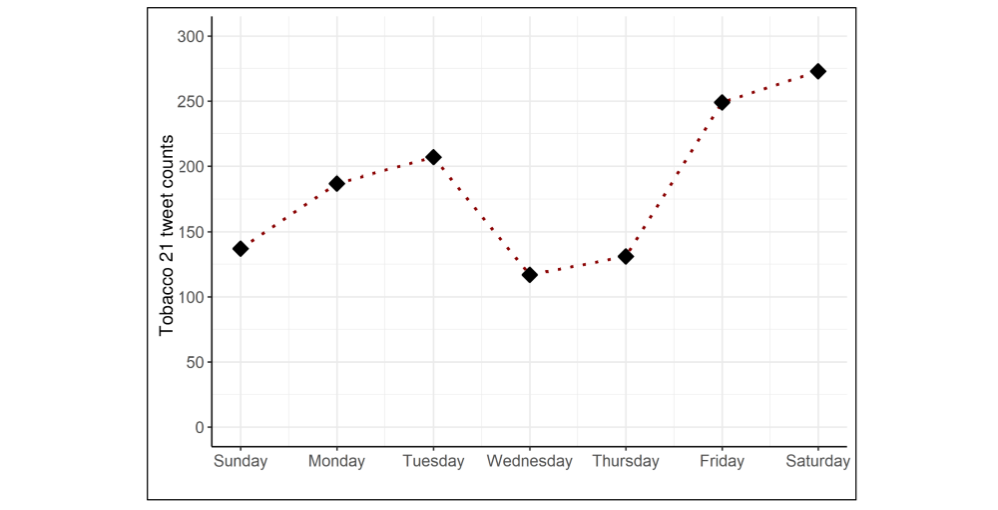
Average number of Tobacco 21 relevant tweets per day of the week.

Of the 4628 tweets from the 2% subsample, 1301 (28.1%) tweets discussed Tobacco 21 specifically. Unrelated tweets were often in response to a tweet about Tobacco 21; however, sentiment was coded based on the most immediate tweet. Among tweets relevant to Tobacco 21, 1113 (85.5%) were relevant to the federal Tobacco 21 law, meaning that no state or local law was mentioned in the tweet. Tweets about Tobacco 21 appeared to be posted most frequently on Saturdays and Fridays (Figure 1); the federal Tobacco 21 policy was enacted on Friday, December 20, 2019.

### Propolicy Sentiment

**Figure 2:**
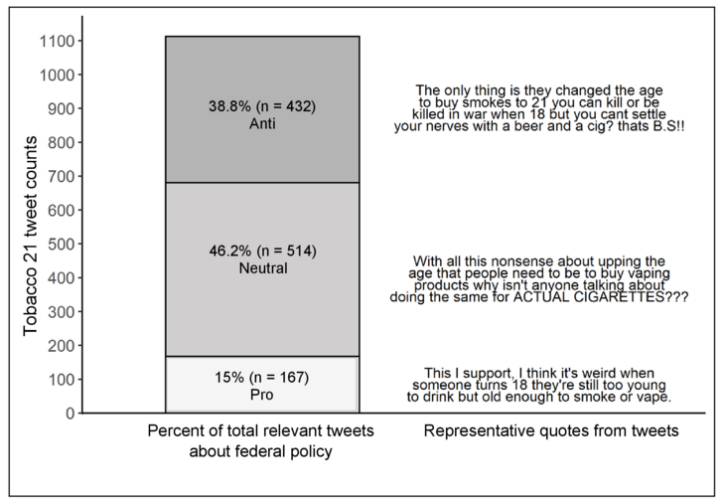
Representative quotes of anti-, pro-, and neutral-sentiment tweets about the federal Tobacco 21 law.

Among the Tobacco 21 tweets, 167 (15%) were supportive of the policy (Figure 2). Themes that emerged from propolicy codes explained that the law represented sensible regulation and protected youth and young adults. Comments from those whose tweets indicated that they vaped and supported vaping endorsed Tobacco 21 as a compromise compared to a complete ban of all vaping products or to flavored vaping products, a regulatory approach feared by some. Some indicated support for Tobacco 21 as a balanced regulatory measure they believed would both reduce youth initiation while also allowing adults to access vaping products. These comments often used phrases that included the word “protection” when describing tobacco and youth. Also, many of these posts provided recommended measures, such as limiting vaping products to vaping or tobacco only stores, raising the MLSA, and limiting nicotine concentrations to <24 mg.

### Antipolicy Sentiment

Overall, 432 (38.8%) tweets clearly opposed the policy (Figure 2). Themes that emerged from the tweets coded as antipolicy included discussions about addiction among those aged 18-20 years and doubt that the policy would reduce smoking. Some discussed repercussions for nicotine-dependent young adults should the law be passed, such as tweets that implied humor (eg, used *lol*) about youth audiences experiencing addiction withdrawals or listed people who might purchase tobacco products for the person underage (eg, parents, friends, and strangers). Other antipolicy sentiments cast doubt that the policy would reduce smoking or included sarcastic remarks criticizing the unimportance of the issue.

### Neutral Sentiment

Overall, 46.2% (n=514) of tweets about the federal Tobacco 21 law were coded as neutral policy, indicating that the tweet was informative rather than opinionated, or that the tone of the tweet was unclear. Many neutral comments connected the Tobacco 21 law to a government entity, political party, or figure. Political party discussion included party affiliations for both democrats and republicans, and many neutral tweets mentioned President Donald Trump explicitly (n=131, 25.5%). Overall, 194 (17.4%) of the federal Tobacco 21 tweets were coded as being reported by a news source or in response to a news article. Most news-related tweets (n=179, 92.3%) were coded as neutral policy. Another emergent theme from neutral tweets was the description of methods of circumventing the law, such as implying that those who were under the MLSA would ask those over 21 to purchase tobacco products for them. After data were recoded, we discovered this theme included mixed sentiment, with 26 tweets representing neutral sentiment (acknowledgement of methods to avoid the law while not implying their policy position) and 42 tweets representing negative sentiment.

### Temporal Trends

**Figure 3:**
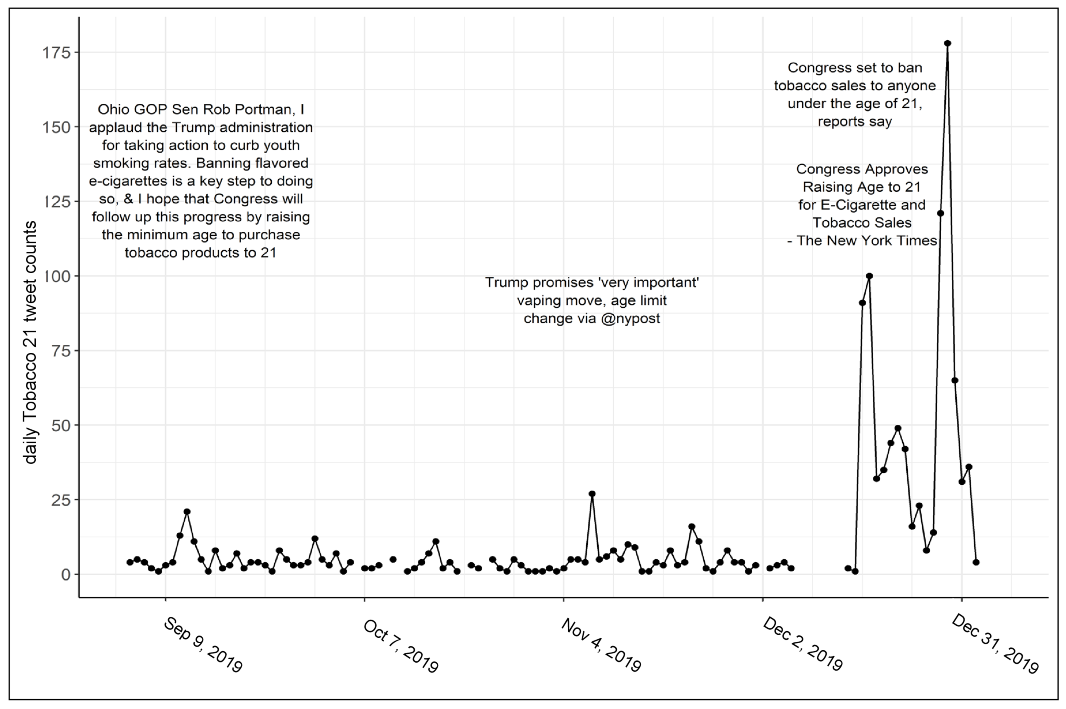
Volume of Tobacco 21–related tweets between September 4, 2019, and January 1, 2020. Vertical grid lines represent 7-day intervals; breaks in the time series indicate data were not collected due to server disconnection.

News articles about a potential federal Tobacco 21 policy surfaced on Twitter as early as September 9, 2020 (or earlier but outside the data collection window); however, most discussion happened around the time the policy was signed into law (Figure 3). Temporal trends indicated that most news-related tweets coded in this subset of data generated little discussion about Tobacco 21 in the months leading up to enactment. Most Twitter discussion generated from news and general discussion emerged in the final days before enactment.

## Discussion

### Principal Findings

We explored Twitter sentiment and temporal trends of discussions about Tobacco 21 leading up to the enactment of the federal law. While Twitter has been used in the past to explore miscommunication about Tobacco 21 [25] and connections to other age-related behaviors [26], our study is unique in its mixed-methods exploration of sentiment about Tobacco 21. We found most tweets about the federal Tobacco 21 law, leading up to its enactment in the United States, were either neutral or antipolicy. These findings contrast those of polling studies conducted prior to 2018 [8-11], when many states and localities began passing their respective ordinances and laws.

Data collected from social media networks (eg, Twitter) have been used in prior research to explore a wide range of health topics including vaccinations [43,44], dementia [45], and use of novel tobacco products such as e-cigarettes [20,37,46]. For example, a text-mining study regarding the announcement of the Deeming Act of the Food and Drug Administration (FDA) found many of the 4629 tweets made during or before enactment were not supportive of the FDA’s regulatory authority of e-cigarettes [23]. Consistent with this, over one-third of tweets from our study expressed disapproval of the federal Tobacco 21 law. Some of these tweets suggested the law may increase illegal behavior, such as using fake identification, by those addicted to nicotine. Understanding this sentiment and the concern for young people’s need to engage in illegal behavior emphasizes the importance of cessation programs for young audiences who are dependent on e-cigarettes [47-50]. Some antipolicy tweets cast doubt on the efficacy or importance of the law used using sarcasm. While this context can be difficult for coders to capture, it was a strength of our study that we used human annotation, given the challenges of training algorithms to do this [29].

Among neutral discussions about Tobacco 21 on Twitter emerged themes that included news articles that discussed the law and references to political figures, parties, and government affiliation. Many of these tweets appeared to be a call to action to the US president at the time; however, the legislative process for amending the federal MLSA relied on an act of congress before the president could sign the law. This may suggest that government agencies could provide education to the public about the regulatory processes or limitations of these agencies (ie, FDA) or people (ie, president) for certain regulatory actions (eg, raising the MLSA vs restricting the sale of flavored tobacco products).

Twitter discussions that supported Tobacco 21 believed it represented a balanced approach to sensible tobacco regulation. This theme framed Tobacco 21 as a better approach than a total ban of all e-cigarette products or on e-cigarette flavors, which was feared among e-cigarette users and vape shop owners during the 2019 e-cigarette and vaping related lung injuries (EVALI) outbreak in the United States [51,52]. This provides more context for policy support consistent with polling studies that suggested most youth, young adults, cigarette users, and adults supported Tobacco 21 laws [8-11,53]. Given that disinformation about Tobacco 21 on Twitter described it incorrectly to be a purchase law (penalizes youth purchasers) instead of correctly as a sales law (penalized retailers) [25], education for retailers and the general public could capitalize on the vaping communities’ policy support by describing it as a well-balanced regulatory approach while also ensuring retailers’ liability for tobacco sales. Further, we found prosentiment to include text that described protecting youth and young adults. This context may have been particularly important for California voters reading public sentiment on social media, provided that Twitter discussions supporting California’s tobacco tax (that ultimately failed to pass a public vote) was criticized for not connecting the issue to young audiences [24]. Although we only found this sentiment reflected in 46 (4%) of the annotated tweets, similar to this previous study about California’s policy initiative [24], messages about Tobacco 21 may have been easier to connect to youth than a tax on tobacco products.

During a similar time frame as our study, news posts about the EVALI were increasing, which have been associated with a concurrent search for e-cigarette cessation resources [54]. Our search indicated small peaks of discussions about Tobacco 21 that occurred around the same time of the news articles about EVALI (September and November) [54]; however, most discussion about the policy occurred on Twitter around the time the law was passed. Thus, the public may have been distracted by broader conversations about the future of e-cigarettes during the EVALI outbreak and may not have been aware of the political movement for this particular legislation. This awareness is important to understand to inform implementation, enforcement, and communication after such laws are enacted. Lack of awareness about this law would suggest a need for an educational campaign and training for retailers and the public alike. Implementation scientists could examine how Tobacco 21 was rolled out to state and local agencies, along with retailers and general public awareness and compliance with the federal law. Moreover, understanding public response also may be of particular interest to policymakers in other countries currently considering raising their legal sales age of tobacco [55].

Prior research has examined cyclical trends for illness (eg, strokes occur most commonly on Mondays) [56] and smoking cessation seeking behaviors (eg, there are 25% more internet searchers for cessation resources on Mondays than all other days of the week combined) [57]. Understanding weekly rhythms of web-based discussions about policies can inform best times to post information about a health topic or respond to Twitter chatter discussing a policy. We found most tweets were posted on Fridays and Saturdays, which differ from the rhythm cycles of other health discussions [56,57]. This may be a response to news generated later in the week, or this timing could be attributed to the enactment of the Tobacco 21 law. Congress passed the bill (H.R. 1865) on Thursday, December 19 and the president signed the bill into law on Friday, December 20, 2020. Thus, research examining time-stamped discussions about the legislative progress of the bills should consider that such data could demonstrate a 1-day delay.

### Limitations

Twitter provides an open platform for public opinion. Therefore, it may capture a vocal audience that does not precisely represent US demographics. Further, search terms used to identify tweets relevant policy evolve over time as public discussion changes. For example, we used *McConnell* as a search term due to Mitch McConnell’s involvement with the first Tobacco 21 bill proposed to the Senate; however, it is unlikely that the name would remain a key search term after the enactment of the policy. Thus, those seeking to explore discussions about Tobacco 21 after enactment of the federal law should consider search terms relevant to the law during the time frame they are exploring. Another limitation of our study was that only 1301 (28.1%) of the identified tweets were relevant to the discussion. There was substantial discussion about tobacco products during the time the data for this analysis were collected [54]. This may have generated broad discussion about tobacco products that included necessary search terms for our data collection but unfortunately captured Twitter chatter that did not discuss Tobacco 21 specifically. An unanticipated disruption of the RITHM data server occurred in our final month of data collection and lasted about a week. Rather than terminating the study early, we maintained consistency with the original research protocol and reengaged with data collection through the 18 remaining days of the calendar year. This nonetheless remains a limitation of our study and may have had a marginal impact on our findings. Further, some tobacco-related policy discussions may include mixed sentiment, which was not annotated in this study but was identified following annotation. Given the complexity of public discussion about tobacco control policies, we recommend for future social media–based research about regulatory processes, capture mixed sentiment as well as sentiment subcategories, such as sarcasm.

Social media–based research may help complement traditional methodologies in identifying awareness of and support for or against tobacco control laws and communities’ readiness for such laws. Further, this research methodology may help researchers provide policymakers with timely and meaningful recommendations for further policy amendments or educational practices. As tobacco access laws (eg, legal sales age and flavor restrictions) are introduced, it will be important to understand communities’ acceptance of such laws to ensure that they are implemented and enforced as intended. Given that most discussion about Tobacco 21 occurred in the final days of enactment, tobacco regulatory agencies should allocate resources for education and compliance for retailers and the public about the law to help address enforcement challenges.

## Abbreviations

EVALI: e-cigarette and vaping related lung injury
FDA: Food and Drug Administration
MLSA: minimum legal sales age
RITHM: Real-Time Infoveillance of Twitter Health Messages

## Data Availability

Data will be made available upon request.
